# Phosphogypsum as Fertilizer: Impacts on Soil Fertility, Barley Yield Components, and Heavy Metals Contents

**DOI:** 10.3390/plants14010016

**Published:** 2024-12-25

**Authors:** M Barka Outbakat, Moussa Bouray, Redouane Choukr-Allah, Mohamed El Gharous, Kamal El Omari, Khalil El Mejahed

**Affiliations:** 1 Agricultural Innovation and Technology Transfer Center (AITTC), Mohammed VI Polytechnic University, Ben Guerir 43150, Morocco; moussa.bouray@um6p.ma (M.B.); redouane.choukrallah@um6p.ma (R.C.-A.); mohamed.elgharous@um6p.ma (M.E.G.); 2 College of Sustainable Agriculture and Environmental Sciences (CAES), Mohammed VI Polytechnic University, Ben Guerir 43150, Morocco; 3 OCP S.A., Sustainability & Green Industrial Development (SGID), Casablanca 20200, Morocco; elomari@ocpgroup.ma

**Keywords:** phosphogypsum, fertilization, yield parameters, nutrients uptake, heavy metals, soil

## Abstract

According to the FAO, 828 million people were facing acute food insecurity in 2021. Fertilization is a critical input factor in crop production and food security achievement. Therefore, fertilization is a critical input factor in crop production and food security achievement. However, there is room for improvement in the application of fertilizers in certain regions. Thus, new fertilizers with a relatively low cost could enhance farmers’ access to these essential inputs. Phosphogypsum (PG) is used as fertilizer because it contains many nutrients essential for plant growth, including calcium, sulfur, and phosphorus. A two-year field experiment was conducted using two Moroccan PG products (PG1 and PG2, obtained from two different industrial sites), applied at four rates (0, 1.5, 3, and 4.5 t/ha). The aim was to assess the impact of PG source and rate on barley crops, including yield component, nutrients uptake, and heavy metals content. The study’s findings revealed that as the rate of PG application increased, there were significant enhancements in the number of spikes, tillers, grains, total biomass, grain yield, and thousand-grain weight. In fact, when compared to the control, the application of 1.5, 3, and 4.5 t/ha of PG led to a remarkable increase in grain yield by 21%, 34%, and 39%, respectively. Furthermore, the uptake of nutrients (N, P, K, Ca, Mg, and S) by the shoots and grains was significantly influenced by the PG application rates, with higher rates resulting in greater nutrient uptake. Notably, the application of PG had no discernible impact on the heavy metal content in shoots, grains, or soil.

## 1. Introduction

Addressing the ongoing challenge of supplying adequate food to every person is among the paramount issues confronting humanity today. According to FAO (2022), 828 million people suffered from hunger in 2021, which means that one in nine people is hungry globally. Furthermore, one in every four people in sub-Saharan Africa is malnourished [1]. Simultaneously, it is anticipated that the growth in both population and income will necessitate a 70% rise in worldwide food production by 2050, with the figure potentially reaching up to 100% in developing nations [2]. Poverty, conflict, climate fluctuations, and often, insufficient agricultural productivity are contributing factors to undernourishment [3]. Consequently, boosting agricultural production is a fundamental approach to attain food security. Numerous studies have explored the extent to which nutrient inputs contribute to crop production. Nelson affirmed the significance of fertilization as an input factor, leading to a 30 to 50% boost in grain crop yields [4]. In the 1970s and 1980s, the increased use of fertilizers accounted for one-third of the global rise in cereal production and half of the grain production increase in India. In sub-Saharan Africa, for instance, the average fertilizer application per hectare of cultivated land stands at a mere 17 kg, in contrast to the worldwide average of 135 kg [5]. In Morocco, the quantity of fertilizers applied only meets 36% of the actual requirements. This situation can be attributed to various factors that encompass the expenses associated with both fertilizers and their application. Consequently, the introduction of cost-effective fertilizers has the potential to enhance farmers’ access to these essential resources, particularly in African regions.

Phosphogypsum is a low-cost co-product of the phosphoric acid industry [6] that is produced in several African countries such as Morocco, Tunisia, South Africa, Egypt, and Senegal [7,8,9,10,11]. Phosphogypsum finds extensive application in agriculture as an amendment for depleted soils (including saline, sodic, alkaline, and acidic) and as a fertilizer [12]. Phosphogypsum contains a variety of essential nutrients crucial for plant growth and development, such as calcium (Ca), sulfur (S), and phosphorus (P). Consequently, it has the potential to serve as a highly effective compound fertilizer [13]. Numerous studies have demonstrated that the utilization of phosphogypsum (PG) leads to enhanced crop yields. For instance, applying 2.3 t/ha of phosphogypsum resulted in yield increases of 6% for tomatoes, 19% for potatoes, and 49% for watermelons compared to untreated soils [14]. Furthermore, our earlier research has shown that applying 30 and 45 t/ha of phosphogypsum for the reclamation of saline soil resulted in a 52% and 62% increase in faba bean grain yield, respectively, in comparison to untreated saline soil with an electrical conductivity (Ece) of 11.2 mS/cm [15]. Moreover, [16] confirmed that the application of 3 t/ha of PG improved the yield of spring chickpeas, winter chickpeas, and lentils, by 50, 30 and 27%, respectively, compared to the control. Additionally, the combination of phosphogypsum (PG) with lime led to a notable rise in lucerne crop yield and the uptake of phosphorus (P) and sulfur (S) by plants in acid soils [17]. Combining phosphogypsum with lime can also enhance the levels of potassium (K), Ca, magnesium (Mg), nitrogen (N), and S in acidic soils [18]. It is also well-established that PG enhances the physical characteristics of the soil. For instance, in a study by [19], it was observed that the application of PG improved soil stability and porosity.

Over the last few decades, the valorization of PG has been of high interest to scientists, engineers, and fertilizer industries [20]. The linear economic “take–make–dispose” process is not a sustainable approach and must be replaced by a circular economy model that converts goods at the end of their useful life into resources for other purposes, thus closing the loop in the industrial ecosystems and reducing the wastes [20,21]. As such, the valorization of phosphogypsum (PG) should be aligned with the principles of the circular economy model. This approach holds great promise for generating added value while diminishing the need for ground storage and sea disposal, thereby reducing its environmental impact.

Phosphogypsum valorization must consider its chemical composition, particularly impurities that could pose environmental concerns. The concentrations of radionuclides and heavy metals in PG vary considerably depending on the source of the rock phosphates as well as the extraction processes [7]. The environmental and sanitary impacts are the main issues regarding its agricultural use. A thorough investigation of these impacts requires long-term studies to monitor soil, plant, and water quality based on heavy metal and radioactive element contents. Long-term studies are essential for capturing the dynamics of trace elements and their interactions in soil–plant–water systems. This information is crucial for developing or refining guidelines.

Morocco holds a significant position as a global producer of phosphogypsum (PG). Substantial efforts have been dedicated to enhancing its utility across various sectors, particularly agriculture. Nonetheless, there remains a scarcity of information concerning the agronomic performance of Moroccan PG, particularly its behavior in soil under field conditions. Additionally, it is essential to recognize that PG properties are heavily influenced by its source, typically rock phosphate, and the industrial processes involved. Consequently, comparing different PG sources could yield valuable insights and aid in the selection of the most effective products. This paper sought to accomplish two main objectives.

1. Evaluate the effects of two Moroccan PG sources on soil fertility and barley yield components under irrigated field conditions.
2. Assess the environmental and health implications of these PG sources on soil, as well as barley shoots and grains, by examining their heavy metals contents.

## 2. Materials and Methods

### 2.1. Trials Installation and Treatments

**Figure 1:**
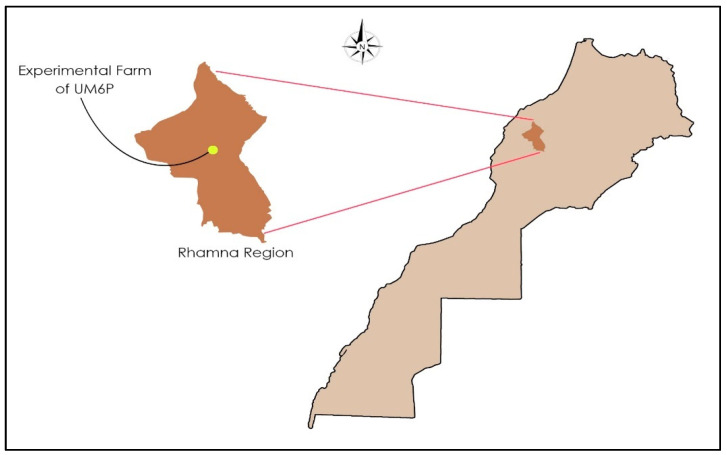
Field trial location.

A two-year experiment (from January to June 2021 and 2022) was conducted at the experimental farm of Mohammed 6 Polytechnic University (UM6P) in Ben Guerir (Figure 1), located in the Rhamna region of central–west Morocco at latitude 32° 13′6′′ North and longitude 7° 52′43′′ West. This region is characterized by an arid Mediterranean climate: cold winters, low precipitation, and dry summer periods [22]. The mean annual rainfall is approximately 168 mm/year [23]. The maximum temperature in summer is 48 °C and the minimum in winter is 3.6 °C [24].

The treatments consisted of two Moroccan PG products (PG1 and PG2, obtained from two different industrial sites), applied at four rates (0, 1.5, 3, and 4.5 t/ha). The PG was applied manually, followed by incorporation into the top 15 cm of the soil using a cover crop, then barley (*Hordeum vulgare* L.) seeds were sown directly in the soil with a seeder at a seeding rate of 90 Kg/ha. A drip irrigation system was used for an automatic water supply to the plants and the irrigation dose was calculated based on the climatic demand and crop characteristics according to Equation (1) developed by the FAO (1998):

ETM = Kc × ETp(1)

where ETM: maximum evapotranspiration, which presents the crop water requirement in mm. ETp: potential evapotranspiration (in mm) measured daily in the weather station installed at the experimental site, and Kc: cultural coefficient that allows correcting the ETp according to crop type and its development stage.

During 2021 and 2022, the plots received a total irrigation water of 329 and 297 mm and a total rainfall of 101 and 72 mm, respectively. A total of 30 kg/ha of nitrogen was supplied through the irrigation water (fertigation), using ammonium nitrate fertilizer, distributed throughout each growing season.

### 2.2. Soil and Phosphogypsum Analysis

Soil samples were collected, at a depth of 0–20 cm, before the establishment of the experiment and after the barley harvesting for each agricultural campaign. Three soil samples were collected using a zigzag design for each experimental plot (3 × 11 m). These three samples were then mixed to obtain one homogenized sample representing each experimental plot. In total, there were 21 experimental plots, which means 63 samples were collected for the entire cultivated area during each agricultural campaign. The soil samples were air-dried and then passed through a 2 mm sieve. The pH and electrical conductivity of the soil were analyzed according to 1:5 (soil: water ratio). Soil exchangeable cations (Na, Ca, K, and Mg) were extracted by ammonium acetate solution at pH = 7 and analyzed using atomic absorption spectroscopy (Agilent Technologies. 200 Series AA, Santa Clara, CA, USA). Soil sulphate was extracted in 1:5 (soil: water ratio) then measured using the colorimetry method (Agilent Technologies, Cary 60 UV-Vis, Santa Clara, CA, USA). Available phosphorus (Olsen P) was measured as described by Olsen [25]. Soil total nitrogen was determined using the Kjeldahl method [26]. Soil organic carbon was measured according to [27]. After extraction with Aqua Regia and two hours of digestion at 95 °C, the soil heavy metals contents were quantified using ICP-OES (Agilent Technologies. 5110 ICP-OES, Santa Clara, CA, USA). Table 1 shows the chemical properties of the initial soil. It is an alkaline soil [28], with low fertility based on its organic matter and phosphorus contents [29]. Except for zinc, the soil heavy metal contents were below the allowable limits determined by the WHO.

The phosphogypsum nutrients and heavy metals contents were quantified using extraction by aqua regia followed by two hours of digestion at 95 °C, then determination by ICP. The pH and EC were measured in the 1:5 PG: water extracts. The two products of PG were selected because they are chemically different, with PG1 being richer than PG2 in terms of S, Ca, and P compared to PG2, while the latter is more acidic (Table 2).

### 2.3. Yield Components and Plant Analysis

The number of tillers was determined at the tillering phase. At the maturation stage, three square meters were harvested, for every experimental plot (11 × 3 m), to determine the number and weight of spikes. After the mechanical crushing of the spikes, the grain yield, number of grains per square meter, and the thousand-grain weight (TGW) were assessed. The harvest index (HI) was calculated as follows:

Harvest index (%) = (Grain yield/Total biomass) × 100

The remainder of the experimental pot area (30 m^2^) was also harvested to determine the total biomass, which was then multiplied by the harvest index to calculate the grain yield.

After yield parameters determination, shoots and grains were analyzed for nutrients and heavy metals contents. Total nitrogen in shoots and grains was measured according to the Kjeldahl method [26], while total phosphorus, potassium, calcium, sulfur, magnesium, and heavy metals were determined using ICP-OES (Agilent Technologies. 5110 ICP-OES, Santa Clara, CA, USA) after extraction with nitric acid.

### 2.4. Statistical Analysis and Experimental Design

The experimental design employed for this study was a split plot with three replicates. The main plots represented the phosphogypsum sources and the subplots represented the different application rates.

Yield components, nutrients uptakes, and heavy metals contents were subjected to analysis of variance (ANOVA) using IMB SPSS (Version 20, IBM SPSS Inc., Chicago, IL, USA). Analysis of variance (ANOVA) was carried out to test the main effect of PG source and rate and the year on the investigated parameters. Prior to conducting ANOVA, the data were tested for normality (using the Shapiro–Wilk test) and homogeneity of variance (using Levene’s test) to meet the assumptions required for the analysis.

If the differences between treatments were significant (*p* < 0.05), the Tukey’s honest significant difference (HSD) was then used as a post hoc test for means separation.

## 3. Results

### 3.1. Climatic Conditions During the Growing Seasons 2021 and 2022 at the Experimental Site

**Figure 2:**
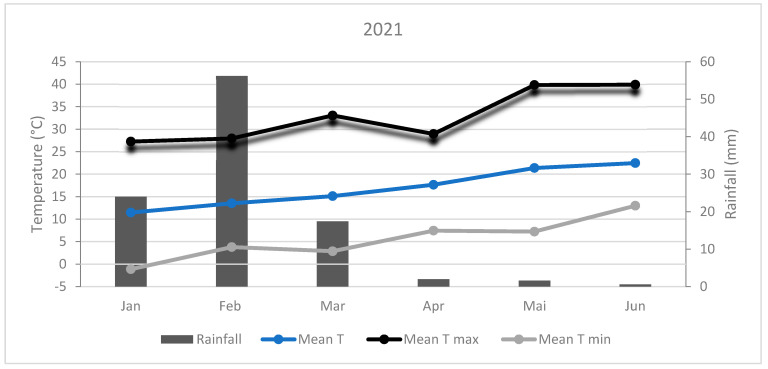
Climatic conditions during 2021.

**Figure 3:**
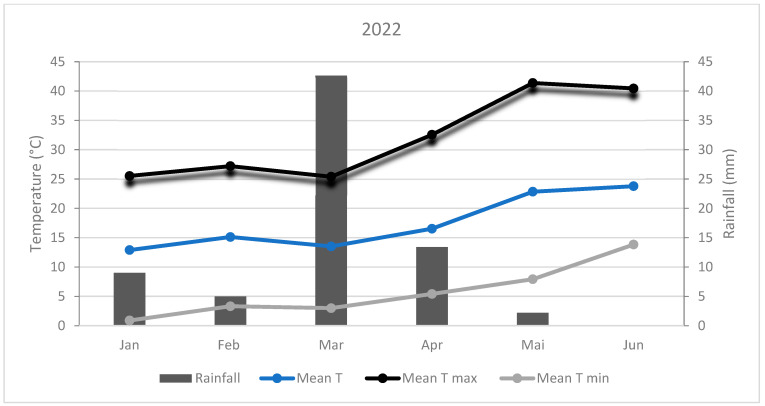
Climatic conditions during 2022.

The experiment site climate is typical of Morocco’s arid regions (Figure 2 and Figure 3). During 2021, the absolute minimum temperature was −1.12°C recorded in January, and the maximum temperature was 39.9 °C recorded in June. However, during the 2022 season, the minimum temperature was 0.89 °C registered in January and the maximum temperature was 41.4 °C recorded in May. The sum of rainfall received was 101.8 and 72.2 mm in 2021 and 2022, respectively. In 2021, the highest amount of precipitation (56.2 mm) was received in February, while in 2022, it was received in March (42.6 mm).

### 3.2. Yield Components

The number of spikes and tillers, as well as the thousand-grain weight, were significantly affected by the PG source and rate and the year (Table 3). These parameters increased as PG rates increased. For instance, during the first season (year 2021), the number of spikes increased by 8, 14, and 26% under 1.5, 3, and 4.5 t/ha of PG regardless of the source, respectively, compared to the control (0 t/ha). However, in the second season (year 2022), these improvements were slightly higher at 15, 20, and 26%, respectively. In addition, PG1 increased the number of tillers and spikes by 14 and 7%, respectively, compared to PG2 across rates. The thousand-grain weight increased by 23% in 2022 compared to 2021. Furthermore, the application of PG at rates of 1.5, 3, and 4.5 t/ha resulted in TGW increases of 3%, 4%, and 6%, respectively, compared to the control.

The number of grains per spike and harvest index were significantly increased by 15 and 34%, in 2022 compared to 2021. However, the PG rate and source had no effect on these two parameters. For each agricultural campaign, the harvest index remained unchanged regardless of the PG rate or source.

There were no interactions between the investigated factors for most of the parameters, except source × rate for the number of tillers and source × year for the number of tillers and TGW. The PG rate and the year had a significant effect on the number of grains, total biomass, and grain yield. Nevertheless, they were not affected by PG sources. The number of grains was significantly influenced by the PG application rate, showing a consistent increase as the PG rate increased. Specifically, applying 1.5, 3, and 4.5 t/ha of PG resulted in increases of 17%, 28%, and 32%, respectively, compared to no PG application. (Table 3).

**Figure 4:**
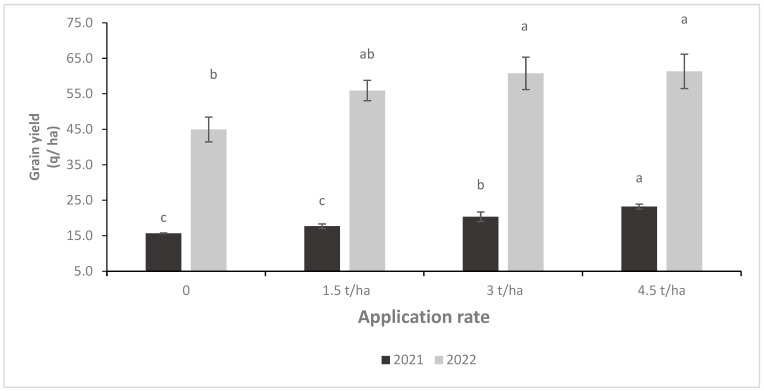
Effect of phosphogypsum rate on barley’s grain yield during 2021 and 2022 seasons. Letters indicate the statistical differences between the effects of PG rates within each year separately. Error bars represent the standard error.

**Figure 5:**
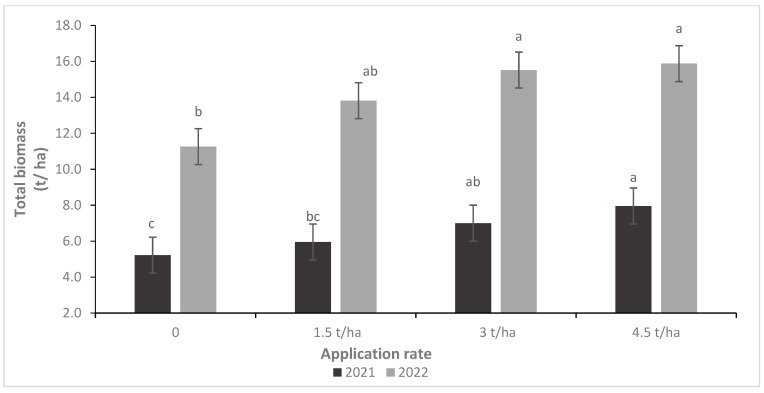
Effect of phosphogypsum rate on barley’s total biomass during 2021 and 2022 seasons. Letters indicate the statistical differences between the effects of PG rates within each year separately. Error bars represent the standard error.

Barley yields in 2022 were significantly higher than in 2021. During 2022, total biomass and grain yield increased by 116% and 190%, respectively, compared to 2021 across rates and sources (Figure 4 and Figure 5). Likewise, the PG rate had a significant effect on total biomass and grain yield. For instance, the total biomass increased by 20, 37, and 45%, and grain yield by 21, 34, and 39% under 1.5, 3 and 4.5 t/ha, respectively, compared to the control, across sources and years.

### 3.3. Nutrients Uptake

#### 3.3.1. Shoot Uptake

Nutrients uptake (kg/ha) was significantly affected by PG rates. For instance, in 2021, shoot nitrogen uptake increased by 3, 20, and 47% at 1.5, 3 and 4.5 t/ha, respectively, across sources, compared to the control (Table 4). Moreover, during 2022, shoot phosphorus uptake increased by 32, 57, and 69% at 1.5, 3 and 4.5 t/ha, respectively, across sources, compared to the control. Except for phosphorus, all nutrient uptakes were affected by the year. Shoot K, Ca, Mg, and S uptakes increased by 94, 83, 85 and 22% during 2022 compared to 2021 across PG rates and sources. Conversely, shoot nitrogen uptake was higher by 80% during the first growing cycle than it was during the second one across PG sources and rates.

**Figure 6:**
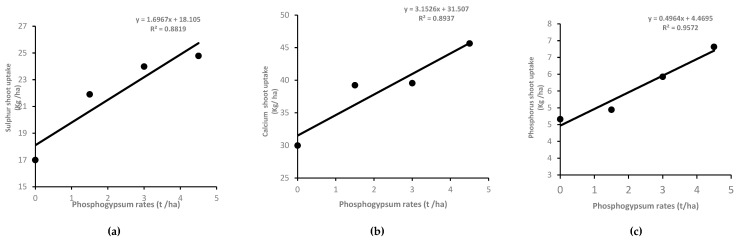
The relationship between phosphogypsum rate and shoot nutrients uptakes. Data points represent the mean of the two growing seasons in 2021 and 2022.

Nitrogen and calcium shoot uptakes were affected (*p* < 0.05) by PG source; in fact, PG1 increased their uptakes by 16 and 30%, respectively, compared to PG2 across years and rates (Table 4). Shoot nutrients uptakes were highly positively correlated with phosphogypsum application rate. Figure 6 shows the linear regression between PG application rate and shoot nutrients (P, S and Ca) uptakes, with high coefficients of determination R2 at 88.2, 90, and 95.8%.

#### 3.3.2. Grains Uptake

Grain nutrient uptakes were significantly affected by the rate of PG and the year, while no effect has been observed for PG source on grain nutrient uptake, except Ca (Table 5). For instance, phosphorus uptake increased by 53, 77, and 95% at 1.5, 3 and 4.5 t/ha, respectively, compared to the control, across years and sources. Furthermore, sulfur uptake increased by 160% in 2022 against 2021 across PG rates and sources. On the other hand, grain Ca uptake increased 100% for PG2 compared to PG1 across years and rates. The interactions between all the investigated factors (PG rate, PG source, and the growing season) were significant for Ca, while only the rate × year interaction effect was significant for rest of the nutrients, specifically K, Mg, and S.

### 3.4. Heavy Metals Contents

Heavy metals contents in shoots were not affected by the PG rate, except for Zn (Table 6). However, the contents of B, Ba, Mn, and Zn in shoots were affected (*p* < 0.001) by the year. No effect has been observed for the PG source on heavy metals contents (Table 6).

Heavy metals contents in grains were not affected by PG rate, except for B, while the PG source affected Cu and Zn contents only (Table 7). The contents of Cu and Zn increased by 10 and 6%, respectively, under PG2 against PG1. Moreover, a significant effect of the year was recorded for B, Ba, Mn and Zn, for which the contents were noticeably higher during the second year (2022) compared to the first year (2021).

The concentrations of Mo, Pb, Sb, and Co in barley shoots and grains were also analyzed, and their concentrations were less than 1 ppm. However, the concentration of Cd was less than the quantification limit of the ICP-OES which is 0.3 ppm in our case.

### 3.5. Soil Parameters

#### 3.5.1. Soil pH and Nutrient Content

Phosphogypsum rate had a significant effect on soil pH and salinity. Increasing PG rates raised soil salinity. The highest value of soil salinity was obtained with 4.5 t/ha of PG. The year significantly affected phosphorus, sulfur, sodium, calcium, and magnesium soil contents, which were higher in the second agricultural campaign compared to the first. Potassium, sodium, calcium, magnesium, and nitrogen were not affected by PG rates and sources (Table 8).

**Figure 7:**
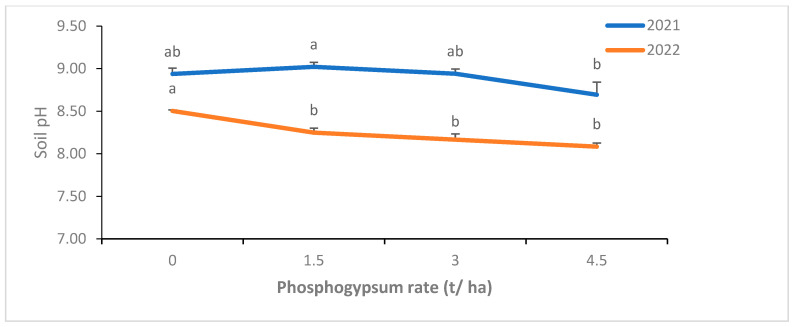
Effect of phosphogypsum rates on soil pH at the end of each season (2021 and 2022). Letters indicate the statistical differences between the effects of PG rates within each year separately. Error bars represent the standard error.

**Figure 8:**
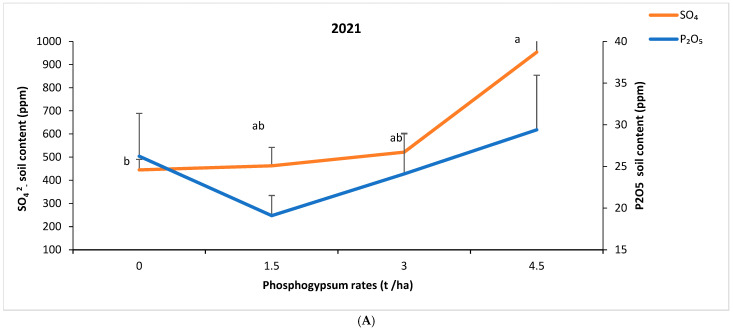

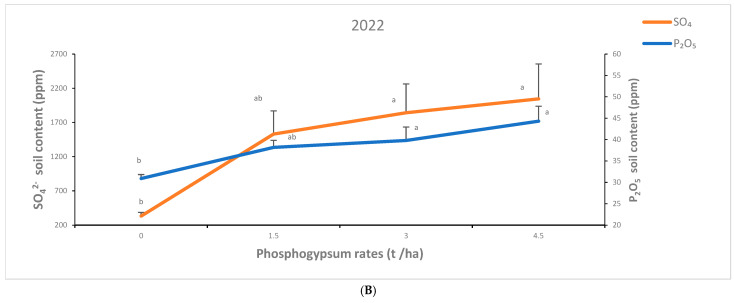
Effect of phosphogypsum rate on soil nutrient contents (**A**): P_2_O_5_ and SO_4_^2−^-2021, (**B**): P_2_O_5_ and SO_4_^2−^-2023. Letters indicate the statistical differences between the effects of PG rates. Error bars represent the standard error.

Increasing the PG rate resulted in a decrease in soil pH and most important pH decrease was observed at 3 and 4.5 t/ha of PG regardless of the source (Figure 7). The phosphorus and sulfur soil contents were significantly enhanced by PG application, especially with the highest PG rate (Figure 8). For instance, compared to the control, 4.5 t/ha of PG increased soil phosphorus content by 12 and 42%, in 2021 and 2022, respectively.

#### 3.5.2. Soil Heavy Metals Contents

Soil heavy metals contents were not affected by PG rate or source. However, the year affected significantly the content of Al, B, Ba, Cd, Co, Mn, Pb, and Zn in the soil. The concentration of heavy metals in the second season (2022) exceeded that of the first (2021), except for Zn (Table 9).

Cadmium soil contents were below the allowable limit in 2021. However, they exceeded that limit in 2022 irrespective of the PG rate and source. Moreover, the concentrations of Zn exceeded the allowable limit for the two years. However, the concentration of Pb and Cu were below the allowable limits (Table 9).

## 4. Discussion

In the present study, PG increased substantially barley yield components compared to the untreated soils. This is in line with previous research works where PG application was found to enhance the yield of many other crops such as tomatoes, potatoes, watermelons [14], chickpeas, lentils, corn, faba beans [19], wheat [31], and sugarcane [32]. Moreover, Li and Chang (2018) found that the PG application promoted rice stem development, reduced water lodging, and increased cotton vegetative development.

Additionally, the resulting improvement of barley grain and shoot nutrients uptakes under PG application is in agreement with previous studies where PG increased S, Ca, N, P, K, and Mg uptake in wheat shoots and grain [33] as well as S and P uptakes in lucerne [17]. The increased yield and nutrients uptake following PG application can partly be attributed to soil nutrients enrichment, as shown in Table 8 and Figure 8. This agrees with [34,35] who reported an increase in soil S, Ca, P, and Mg contents after PG addition to different soil types. This suggests that the use of co-products such as PG, which is particularly rich in S, could be a promising way to overcome at least the S deficiency-related problems (yield and quality) and the cost associated with the use of expensive S-based fertilizers. This is evidenced by the fact that sulphate (SO_4_^2−^) concentration in our soils increased three times under PG regardless of the source and year, as compared to the untreated soils. Furthermore, gypsum application has been documented to diminish nutrient loss in the soil, notably P and N [36]. Consequently, this facilitates their availability to plants, resulting in improved plant nutrition and enhanced photosynthetic efficiency [37].

Soil pH is among the main factors that affect nutrients absorption and solubility, especially in alkaline soils such as ours where pH is high (8.6), leading to low nutrient availability [38]. Therefore, the resulting significant decrease in soil pH after a 2-year application of PG might have contributed to boosting nutrients availability in our soils. This could explain, to some extent, the resulting enhancement of shoot and grain nutrients uptake and yield components of barley in this study. On the other hand, soil biological activity is also crucial for nutrient cycling and bioavailability, and therefore plant biomass production (yield). Interestingly, PG has been found to improve soil microbial activity [39]. Nevertheless, this aspect remains unexplored in the present study, and further investigations are necessary to gain a comprehensive understanding of how PG impacts soil biology and its subsequent implications for plant nutrition and soil health.

The comparison between two PG sources revealed that PG1 performed better than PG2 in enhancing the number of spikes and tillers, thousand-grain weight, and shoot nitrogen and calcium uptake. This could be due to the fact that PG1 contains a high amount of Mg, Ca, S, and P compared to PG2 (Table 2).

In general, yield components and nutrient uptake by shoots and grains were higher in 2022 compared to 2021. This can be attributed primarily to the prevailing weather conditions. In 2021, the germination phase experienced cold temperatures, with recorded values of −1.12 °C for the mean minimal temperature and 11.48 °C for the mean temperature, in contrast to the milder conditions of 0.89 °C and 12.9 °C in 2022. Research conducted by [40] has confirmed that lower temperatures have a detrimental effect on the germination of barley seeds. Additionally, it is worth noting that in 2021, the cultivated variety was Najah, whereas in 2022, it was Amalou. Najah is a Spanish variety with low adaptation to Moroccan conditions. However, Amalou is a Moroccan selection more adapted to arid and semi-arid regions; it is a highly productive variety [41].

The findings of this study indicate that the application of PG did not have a significant impact on the levels of heavy metals in the plant shoots, grains, or soil, regardless of the source and application rate. This is indeed interesting information. Nevertheless, conducting long-term investigations is imperative to thoroughly assess the sanitary and environmental consequences of repeated phosphogypsum application using different soil types and crops. Our results are in line with [42], who conducted field trials using Kochia scoparia as a forage crop with a PG application of 10 to 40 t/ha. They concluded that PG had no effect on heavy metals accumulation either in soil or in plants, except fluoride, which increased in the plant but without exceeding the allowable limit. However, fluoride was not analyzed in the present study.

The absence of significant effects on heavy metal content in shoots, grains, or soil, despite the variable doses of phosphogypsum, can be attributed to several factors. First, the studied soil is alkaline, and its high pH negatively correlates with heavy metal solubility and transfer to plants. Second, the soil’s heavy metal contents (before PG application: Table 1) were below the allowable limits set by the WHO, reducing the likelihood of detectable changes. Lastly, the application of phosphogypsum was limited to a relatively short period of two years, which may not have been sufficient to observe long-term effects. Conducting a more extended experiment could provide deeper insights into the impact of different application rates.

In a study conducted by [43], it was demonstrated that even with higher PG application rates ranging from 0 to 200 t/ha, the use of phosphogypsum led to a decrease in the concentrations of Mn, Co, and Cu in tomato shoots, as well as a reduction in the levels of B, Cu, Sb, Cs, Ba, Tl, and Th in tomato fruits. Simultaneously, it resulted in increased accumulation of Se and Cd in the fruits. In another investigation by [44], it was reported that phosphogypsum application did not result in soil pollution with Zn, V, Cr, Ni, and Pb. However, there was a slight contamination observed due to Cd. Furthermore, the authors of the same study confirmed that the concentrations of Zn, Cd, Pb, and Cr in tomato and green pepper fruits remained below the permissible limits for food production. This aligns with our earlier findings obtained from experiments conducted in pots using faba beans [45]. Long-term application of PG at high rates (20 and 25 t/ha every 2–3 years from 1978 to 2006) increased Cd levels in tomato fruits [46]. However, a three-year field experiment found no statistically significant differences in ^222^Rn exhalation rates between the control and PG-treated plots. The mean exhalation rates remained within the lowest quartile of the typical range for soils [47].

From the results of our study and the literature discussed, it was concluded that phosphogypsum is a safe and efficient fertilizer that effectively increases yield production. Its application has demonstrated clear benefits in improving key agronomic parameters, making it a valuable tool for enhancing agricultural productivity. Specifically, long-term studies are needed under diverse pedoclimatic conditions to thoroughly evaluate its environmental, biological, and agronomic effects. These studies should investigate the potential accumulation of heavy metals, changes in soil properties, and any impacts on water quality and surrounding ecosystems by addressing these factors, future research can provide a more comprehensive understanding of phosphogypsum’s role in sustainable agriculture and its long-term viability as a fertilizer.

## 5. Conclusions

Circular production models are currently a necessity to establish a sustainable agriculture system. The recycling of PG in agriculture emerges a crucial strategy to mitigate its storage and disposal impacts. This research, conducted through a two-year field trial, has demonstrated that utilizing PG as a fertilizer leads to increased crop yields. For instance, the total biomass showed an increase of 20%, 37%, and 45% under 1.5 t/ha, 3 t/ha, and 4.5 t/ha, respectively, compared to the controls, across all sources and years. On the other hand, PG1 was more effective than PG2 in increasing spikes, tillers, thousand-grain weight, and shoot nitrogen and calcium uptake, likely due to its higher Mg, Ca, S, and P content. PG application enhanced nutrient uptake, and improved soil fertility but did not reveal a significant impact on heavy metal content in soil, shoots, and grains. This is particularly valuable information in light of the rising global concerns over nutrient deficiencies and soil depletion. Phosphorus, Ca, and S deficiencies are on the rise worldwide, making PG a promising product for addressing these issues and contributing to food security, particularly in regions such as Africa with low fertilizer usage rates. However, for sustainable use of PG in agriculture, long-term studies under different pedoclimatic conditions are highly needed to profoundly investigate phosphogypsum’s environmental effects (including heavy metals and radioactive elements content), as well as its biological and agronomical impacts.

## Figures and Tables

**Table 1:** Chemical characterization of the initial soil before the establishment of the experiment.

Parameter	Value	Parameter	Value
pH	8.6	Cd (ppm)	0.5
EC_1/5_ dS/m	0.61	Co (ppm)	25
Organic matter (%)	2.3	Cr (ppm)	32
P_2_O_5_ (ppm)	15	Cu (ppm)	22
K_2_0 (ppm)	335	Fe (ppm)	18,409
CaO (ppm)	6011	Li (ppm)	33
Na_2_O (ppm)	1028	Mn (ppm)	446
MgO (ppm)	848	Mo (ppm)	<0.01
SO_4_^2−^ (ppm)	235	Ni (ppm)	27
Total nitrogen %	0.16	Pb (ppm)	23
As (ppm)	28	Sb (ppm)	2
B (ppm)	26	Se (ppm)	<0.01
Ba (ppm)	114	Zn (ppm)	65

**Table 2:** Chemical characterization of the investigated phosphogypsum products.

Parameter	PG1	PG2	Parameter	PG1	PG2
EC dS/m	2.4	4.72	Zn (ppm)	8.52	2.21
pH	5.8	2.78	Mn (ppm)	1.83	1.3
S (%)	24	22	Cr (ppm)	6.33	3.19
Ca (%)	26	24	Mn (ppm)	1.83	1.3
P (%)	0.8	0.4	Li (ppm)	0.66	<0.01
Mg (ppm)	259	103	As (ppm)	1.26	0.84
K (ppm)	869	972	Pb (ppm)	1.93	1.93
Co (ppm)	0.13	0.07	Se (ppm)	0.43	<0.01
Sb (ppm)	0.69	0.84	Mo (ppm)	1.02	0.8
Cd (ppm)	4.71	0.81	Ni (ppm)	1.78	0.57
B (ppm)	21.49	20.79	Cu (ppm)	2.59	2.41

**Table 3:** Yield components of barley as affected by the phosphogypsum source and rate, and the year.

Year	PG Source	Application Rate (t/ha)	Nb of Spikes m^2^	Nb of Tillers/m^2^	Nb of Grains/m^2^	Nb of Grains Per Spike	Total Biomass (t/ha)	Grain Yield (q/ha)	TGW (g)	Harvest Index (%)
2021	PG1	0	201.9	894.2	4221.0	20.9	5.2	15.7	37.2	0.3
		1.5	225.7	1053.8	4915.1	21.8	6.4	18.6	37.9	0.3
		3	240.6	1084.2	5813.4	24.2	7.8	22.3	38.4	0.3
		4.5	256.4	1152.6	6071.7	23.7	8.5	24.3	40.0	0.3
	PG2	0	201.9	894.2	4221.0	20.9	5.2	15.7	37.2	0.3
		1.5	209.8	987.6	4453.1	21.2	5.5	16.8	37.6	0.3
		3	221.4	1007.3	4869.1	21.9	6.2	18.4	37.7	0.3
		4.5	252.1	1094.3	5653.4	22.4	7.4	22.1	39.1	0.3
2022	PG1	0	406.7	1410.7	9911.9	22.7	11.3	44.9	45.3	0.4
		1.5	466.7	1691.6	11,689.3	23.7	13.6	55.1	47.1	0.4
		3	536.7	2652.4	13,443.3	24.6	16.8	64.9	48.3	0.4
		4.5	542.0	2621.3	12,916.1	23.8	15.9	62.5	48.5	0.4
	PG2	0	406.7	1410.7	9911.9	22.7	11.3	44.9	45.3	0.4
		1.5	467.7	1876.7	12,040.2	23.6	14.0	56.8	47.1	0.4
		3	438.7	1543.1	12,099.9	24.7	14.2	56.6	46.7	0.4
		4.5	480.3	2191.1	12,745.5	24.5	15.8	60.1	47.1	0.4
*p* rate	Source		*	*	ns	ns	ns	ns	**	ns
	Rate		***	***	***	ns	***	***	***	ns
	Year		***	***	***	***	***	***	***	***
	Source × Rate		ns	*	ns	ns	ns	ns	ns	ns
	Source × Year		ns	ns	ns	ns	ns	ns	ns	ns
	Rate × Year		ns	*	ns	ns	ns	ns	*	ns
	Source × Rate × Year		ns	ns	ns	ns	ns	ns	ns	ns

TGW: 1000-grain weight, PG: phosphogypsum. *: Indicates a statistically significant difference at the *p* < 0.05 level. **: Indicates a statistically significant difference at the *p* < 0.01 level. ***: Indicates a statistically significant difference at the *p* < 0.001 level. ns: Not statistically significant (*p* ≥ 0.05).

**Table 4:** Shoot nutrients uptake (kg/ha) of barley plants as affected by the phosphogypsum source, rate, and the year.

			(Kg/ha)					
Year	PG Source	Application Rate (t/ha)	N	P	K	Ca	Mg	S
2021	PG1	0	57.5	5.2	87.4	22.4	7.8	18.3
		1.5	66.5	4.9	129.8	26.7	9.5	21.0
		3	74.0	6.4	153.1	32.2	10.7	22.9
		4.5	90.9	7.2	169.2	39.2	12.7	24.1
	PG2	0	57.5	5.2	87.4	22.4	7.8	18.3
		1.5	51.5	4.2	116.4	22.3	7.5	15.9
		3	64.4	4.5	124.2	24.0	8.2	16.9
		4.5	78.5	6.1	146.2	28.8	9.6	20.9
2022	PG1	0	26.1	4.1	198.2	37.6	13.2	15.7
		1.5	42.3	5.2	239.9	56.1	16.6	23.5
		3	54.1	7.1	260.3	66.8	19.7	29.4
		4.5	39.8	6.1	273.0	67.5	19.1	25.3
	PG2	0	26.1	4.1	198.2	37.6	13.2	15.7
		1.5	37.4	5.6	253.5	51.9	17.6	27.3
		3	32.4	5.8	255.3	35.1	16.2	26.7
		4.5	41.9	7.8	286.1	47.1	20.6	28.9
*p* rate	Source		**	ns	ns	***	ns	ns
	Rate		***	***	***	**	***	***
	Year		***	ns	***	***	***	***
	Source × Rate		ns	ns	ns	*	ns	ns
	Source × Year		ns	ns	ns	ns	ns	ns
	Rate × Year		*	ns	ns	ns	ns	*
	Source × Rate × Year		ns	ns	ns	ns	ns	ns

*: Indicates a statistically significant difference at the *p* < 0.05 level. **: Indicates a statistically significant difference at the *p* < 0.01 level. ***: Indicates a statistically significant difference at the *p* < 0.001 level. ns: Not statistically significant (*p* ≥ 0.05).

**Table 5:** Barley grain nutrients uptakes (kg/ha) as affected by the phosphogypsum source and rate and the year.

			(Kg/ha)					
Year	PG Source	Application Rate (t/ha)	N	P	K	Ca	Mg	S
2021	PG1	0	25.9	5.7	9.3	1.0	1.9	2.4
		1.5	33.6	6.3	10.8	1.0	2.1	3.3
		3	41.4	7.6	12.8	1.4	2.5	3.6
		4.5	48.8	8.6	14.2	1.4	2.8	4.0
	PG2	0	25.9	5.7	9.3	1.0	1.9	2.4
		1.5	34.2	5.6	9.5	1.0	1.9	2.7
		3	35.1	6.0	9.9	1.0	2.0	2.9
		4.5	45.4	7.6	12.6	1.4	2.6	3.9
2022	PG1	0	78.1	9.6	27.1	1.8	4.9	5.7
		1.5	100.0	11.3	34.1	1.1	5.9	8.7
		3	115.0	13.8	39.5	2.1	7.1	9.2
		4.5	109.8	14.6	39.6	4.0	7.3	9.6
	PG2	0	78.1	9.6	27.1	1.8	4.9	5.7
		1.5	104.0	11.8	34.2	5.3	6.6	8.5
		3	99.2	12.9	35.8	7.1	6.6	8.8
		4.5	104.5	13.6	37.5	9.7	7.0	9.1
*p* rate	Source		ns	ns	ns	***	ns	ns
	Rate		***	***	***	***	***	***
	Year		***	***	***	***	***	***
	Source × Rate		ns	ns	ns	***	ns	ns
	Source × Year		ns	ns	ns	***	ns	ns
	Rate × Year		ns	ns	*	***	*	**
	Source × Rate × Year		ns	ns	ns	***	ns	ns

*: Indicates a statistically significant difference at the *p* < 0.05 level. **: Indicates a statistically significant difference at the *p* < 0.01 level. ***: Indicates a statistically significant difference at the *p* < 0.001 level. ns: Not statistically significant (*p* ≥ 0.05).

**Table 6:** Trace elements content in barley plant shoots as affected by the phosphogypsum source and rate and the year.

			ppm						
Year	PG Source	Application Rate (t/ha)	A	B	Ba	Cu	Fe	Mn	Zn
2021	PG1	0	126.4	11.4	16.6	4.7	143.7	61.7	19.7
		1.5	107.0	11.4	15.6	4.6	119.5	50.6	29.4
		3	103.2	10.3	13.3	3.3	119.6	50.4	16.0
		4.5	95.5	12.3	15.0	3.8	116.2	52.8	15.4
	PG2	0	126.4	11.4	16.6	4.7	143.7	61.7	19.7
		1.5	69.5	10.3	16.7	4.0	82.6	55.9	17.9
		3	79.8	11.0	14.8	3.9	99.0	50.3	16.4
		4.5	89.4	9.9	13.5	3.8	110.5	53.1	19.0
2022	PG1	0	72.5	12.9	25.5	2.4	111.2	60.0	8.9
		1.5	104.5	14.4	17.6	3.4	140.7	78.1	9.5
		3	100.6	13.9	19.8	2.3	163.9	69.2	6.6
		4.5	72.8	13.4	20.4	2.0	105.9	71.2	5.7
	PG2	0	72.5	12.9	25.5	2.4	111.2	60.0	8.9
		1.5	97.3	14.7	17.1	2.1	180.3	70.3	8.7
		3	72.4	13.5	21.9	1.7	97.0	68.3	6.9
		4.5	95.1	14.3	19.1	2.0	122.1	73.7	7.0
*p* rate	Source		ns	ns	ns	ns	ns	ns	ns
	Rate		ns	ns	ns	ns	ns	ns	*
	Year		ns	***	***	ns	ns	***	***
	Source × Rate		ns	ns	ns	ns	ns	**	ns
	Source × Year		ns	ns	ns	ns	ns	ns	ns
	Rate × Year		ns	ns	ns	ns	ns	ns	ns
	Source × Rate × Year		ns	*	ns	ns	ns	ns	ns
Recommended limit ^a^						73.3	425.5	500	99.4

^a^: FAO and WHO, 1989 [30]. *: Indicates a statistically significant difference at the *p* < 0.05 level. **: Indicates a statistically significant difference at the *p* < 0.01 level. ***: Indicates a statistically significant difference at the *p* < 0.001 level. ns: Not statistically significant (*p* ≥ 0.05).

**Table 7:** Trace elements content in barley grains as affected by the phosphogypsum source and rate and the year.

			ppm						
Year	PG Source	Application Rate (t/ha)	Al	B	Ba	Cu	Fe	Mn	Zn
2021	PG1	0	14.6	1.5	3.4	5.4	47.4	15.1	33.3
		1.5	11.9	1.0	2.0	5.1	33.3	15.4	30.4
		3	9.4	1.0	3.0	5.2	32.6	14.6	29.7
		4.5	11.3	1.0	1.7	5.3	37.0	15.2	29.9
	PG2	0	14.6	1.5	3.4	5.4	47.4	15.1	33.3
		1.5	15.5	1.1	1.8	5.3	42.7	16.0	29.5
		3	6.9	0.9	1.5	4.8	33.6	14.0	29.1
		4.5	13.1	1.1	1.6	5.3	33.0	16.3	29.6
2022	PG1	0	31.8	12.3	2.3	3.7	58.9	18.2	23.0
		1.5	23.2	10.4	1.8	3.7	64.6	18.2	20.4
		3	23.3	10.5	1.9	3.8	64.4	18.4	19.7
		4.5	22.2	10.4	2.0	4.2	74.6	20.0	20.8
	PG2	0	31.8	12.3	2.3	3.7	58.9	18.2	23.0
		1.5	23.3	10.3	1.6	5.5	73.8	17.7	24.6
		3	23.2	10.2	4.7	5.1	73.3	19.0	25.9
		4.5	20.4	10.2	1.9	5.1	65.0	17.9	24.2
*p* rate	Source		ns	ns	ns	***	ns	ns	*
	Rate		ns	***	ns	ns	ns	ns	ns
	Year		***	***	ns	***	***	***	***
	Source × Rate		ns	ns	ns	ns	ns	ns	ns
	Source × Year		ns	ns	ns	***	ns	ns	**
	Rate × Year		ns	***	ns	*	ns	ns	ns
	Source × Rate × Year		ns	ns	ns	ns	ns	ns	ns
Recommended limit ^a^						73.3	425.5	500	99.4

^a^: FAO and WHO, 1989 [30]. *: Indicates a statistically significant difference at the *p* < 0.05 level. **: Indicates a statistically significant difference at the *p* < 0.01 level. ***: Indicates a statistically significant difference at the *p* < 0.001 level. ns: Not statistically significant (*p* ≥ 0.05).

**Table 8:** Soil chemical properties as affected by phosphogypsum source and rate and the year.

Year	PG Source	Application Rate (t/ha)	pH	EC (dS/m)	P_2_O_5_ (ppm)	SO_4_^2−^ (ppm)	K_2_O (ppm)	Na_2_O (ppm)	CaO (ppm)	MgO (ppm)	N%
2021	PG1	0	8.9	0.7	26.2	445.0	275.7	1587.7	7483.3	1381.7	0.1
		1.5	9.1	0.6	17.5	412.3	309.7	1365.0	8334.7	1346.7	0.1
		3	8.9	0.7	25.1	530.7	300.3	1551.3	8328.0	1304.7	0.1
		4.5	8.7	1.0	35.7	985.5	315.8	1474.7	8870.3	1266.3	0.1
	PG2	0	8.9	0.7	26.2	445.0	275.7	1587.7	7483.3	1381.7	0.1
		1.5	9.0	0.7	20.7	513.2	347.8	1640.0	7282.3	1243.3	0.1
		3	8.9	0.7	23.1	512.3	303.6	1485.0	8264.0	1358.3	0.1
		4.5	8.7	0.9	23.1	921.7	340.9	1401.3	6861.7	1248.3	0.1
2022	PG1	0	8.5	0.5	30.9	332.2	359.5	1091.0	7179.7	1514.7	0.1
		1.5	8.3	0.8	38.3	1157.5	422.6	1277.3	8499.3	1412.0	0.1
		3	8.2	0.9	41.6	1651.5	356.0	1133.7	8567.7	1445.3	0.1
		4.5	8.1	1.2	46.7	1907.7	376.1	1203.7	9677.0	1478.0	0.1
	PG2	0	8.5	0.5	30.9	332.2	359.5	1091.0	7179.7	1514.7	0.1
		1.5	8.2	1.0	38.1	1902.0	468.5	1396.7	7568.0	1443.0	0.1
		3	8.1	1.2	38.0	2032.2	378.9	1237.7	8655.3	1396.3	0.1
		4.5	8.0	1.1	41.9	2182.0	329.7	1075.7	8609.0	1357.3	0.1
Source			ns	ns	ns	ns	ns	ns	ns	ns	ns
Rate			***	**	*	***	ns	ns	ns	ns	ns
Year			***	ns	***	***	ns	***	***	**	ns
Source × Rate			ns	ns	ns	ns	ns	ns	ns	ns	ns
Source × Year			ns	ns	ns	ns	ns	ns	ns	ns	ns
Rate × Year			**	*	ns	**	ns	ns	ns	ns	ns
Source × Rate × Year			ns	ns	ns	ns	ns	ns	ns	ns	ns

*: Indicates a statistically significant difference at the *p* < 0.05 level. **: Indicates a statistically significant difference at the *p* < 0.01 level. ***: Indicates a statistically significant difference at the *p* < 0.001 level. ns: Not statistically significant (*p* ≥ 0.05).

**Table 9:** Soil heavy metals contents as affected by phosphogypsum source and rate and the year.

			Trace Elements Concentration in ppm									
Year	PG Source	ApplicationRate (t/ha)	Al	B	Ba	Cd	Co	Cu	Fe	Mn	Pb	Zn
2021	PG1	0	29,864.9	26.5	154.2	0.5	12.6	31.3	36,995.7	456.7	27.0	87.5
		1.5	27,576.4	25.4	143.6	0.6	12.5	31.5	35,215.2	591.9	28.9	87.5
		3	28,939.4	24.4	159.0	0.5	12.9	32.1	35,394.5	664.9	29.6	88.9
		4.5	27,374.3	24.7	146.2	0.6	12.5	32.7	33,939.4	527.5	27.5	87.8
	PG2	0	29,864.9	26.5	154.2	0.5	12.6	31.3	36,995.7	456.7	27.0	87.5
		1.5	29,078.1	26.5	149.9	0.5	12.7	31.1	36,247.6	545.2	29.0	88.3
		3	27,807.8	25.3	142.8	0.5	12.0	30.1	33,929.6	548.2	28.9	86.5
		4.5	28,627.8	27.1	144.2	0.5	12.6	30.0	35,506.1	554.2	25.3	88.6
2022	PG1	0	41,540.2	44.9	157.4	1.2	15.5	31.6	34,531.8	615.1	31.9	84.2
		1.5	41,999.5	43.0	157.6	1.2	15.3	33.1	34,695.1	601.2	28.2	84.2
		3	37,302.3	40.1	147.5	1.2	14.5	31.1	32,545.5	564.4	28.3	79.3
		4.5	38,582.0	42.9	153.3	1.2	14.9	32.7	33,548.4	589.8	29.6	80.2
	PG2	0	41,540.2	44.9	157.4	1.2	15.5	31.6	34,531.8	615.1	31.9	84.2
		1.5	40,630.7	42.4	157.8	1.2	16.3	31.0	35,019.7	631.1	33.6	86.7
		3	41,922.1	45.6	158.2	1.3	18.3	36.1	35,421.5	617.1	34.0	86.7
		4.5	40,938.9	44.1	166.1	1.2	16.0	30.6	33,528.0	622.0	27.3	81.3
Source			ns	ns	ns	ns	ns	ns	ns	ns	ns	ns
Rate			ns	ns	ns	ns	ns	ns	ns	ns	ns	ns
Year			***	***	ns	***	***	ns	ns	*	*	*
Source × Rate			ns	ns	ns	ns	ns	ns	ns	ns	ns	ns
Source × Year			ns	ns	ns	ns	ns	ns	ns	ns	ns	ns
Rate × Year			ns	ns	ns	ns	ns	ns	ns	ns	ns	ns
Source × Rate × Year			ns	ns	ns	ns	ns	ns	ns	ns	ns	ns
Maximum limit WHO						0.8		36			85	50

*: Indicates a statistically significant difference at the *p* < 0.05 level. ***: Indicates a statistically significant difference at the *p* < 0.001 level. ns: Not statistically significant (*p* ≥ 0.05).

## Data Availability

Data is contained within the article.
